# Supplementary material for: An AI-powered Bayesian Generative Modeling Approach for Causal Inference in Observational Studies
Source: J Am Stat Assoc. Author manuscript; Available in PMC 2026 Jul 9. (PMC13344347; doi:10.1080/01621459.2026.2654227)
Supplement: Supp 1 [file NIHMS2185026-supplement-Supp_1.zip › uasa_a_2654227_sm7019.docx]

Author Contributions Checklist Form

This form documents the artifacts associated with the article (i.e., the data and code supporting the computational findings) and describes how to reproduce the findings.

# Part 1: Data

This paper **does not** involve analysis of external data (i.e., no data are used or the only data are generated by the authors via simulation in their code).

I certify that the author(s) of the manuscript have legitimate access to and permission to use the data used in this manuscript.

## Abstract

We used two public datasets in our semi-synthetic studies.

1. Twins dataset. This dataset contains data of 71,345 twins, including their weights (used as treatment), mortality, and 50 other covariates derived from all births in the USA between 1989-1991. The raw data is from <http://www.nber.org/data/linked-birth-infant-death-data-vital-statistics-data.html>. Specifically, we use the files from <http://www.nber.org/lbid/1989/linkco1989us_den.csv.zip>, <http://www.nber.org/lbid/1990/linkco1990us_den.csv.zip>, and <http://www.nber.org/lbid/1991/linkco1991us_den.csv.zip>

2. ACIC2018 data. This data is from the 2018 Atlantic Causal Inference Conference (ACIC) competition, which utilizes the Linked Births and Infant Deaths Database (LBIDD) based on real-world medical measurements. We used 9 datasets from this ACIC2018 with different sample sizes. The UFIDs for the 9 datasets are: 629e3d2c63914e45b227cc913c09cebe, 35524a031525484dab3b06f3728c708e, a957b431a74a43a0bb7cc52e1c84c8ad, 71f29913f174456e9fe2727b1b86b8b3, fda655aeb8644c9db5c543ed9d1006ad, 05fdeea9fcb64b3885e6ebfb85b4ce90, 1c565ac309074f178a377c2759333209, b73beac2f4c349fb981880399d4c88a6, d5bd8e4814904c58a79d7cdcd7c2a1bb

## Availability

Data **are** publicly available

Data **cannot be made** publicly available

If the data are publicly available, see the *Publicly available data* section. Otherwise, see the *Non-publicly available dat*a section, below.

### Publicly available data

Data are available online at: <https://www.nber.org/research/data/linked-birthinfant-death-cohort-data>, <http://www.nber.org/lbid/1989/linkco1989us_den.csv.zip> , <http://www.nber.org/lbid/1990/linkco1990us_den.csv.zip>, <http://www.nber.org/lbid/1991/linkco1991us_den.csv.zip> and <https://www.synapse.org/Synapse:syn11738963>

Data are available as part of the paper’s supplementary material.

Data are publicly available by request, following the process described here:

Data are or will be made available through some other mechanism, described here:

### Non-publicly available data

Discussion of lack of publicly available data:

## Description

### File format(s)

CSV or other plain text:

Software-specific binary format (.Rda, Python pickle, etc.):

Standardized binary format (e.g., netCDF, HDF5, etc.):

Other (described here):

### Data dictionary

Provided by the authors in the following file(s):

Data file(s) is (are) self-describiing (e.g., netCDF files)

Available at the following URL:

Twins raw data: <https://www.nber.org/research/data/linked-birthinfant-death-cohort-data>, <http://www.nber.org/lbid/1989/linkco1989us_den.csv.zip> , <http://www.nber.org/lbid/1990/linkco1990us_den.csv.zip>, <http://www.nber.org/lbid/1991/linkco1991us_den.csv.zip>

Twins processed data:

ACIC 2018 dataset: <https://www.synapse.org/Synapse:syn11738963>

### Additional information (optional)

ACIC 2018 data requires user registration to access and download the data. We use the 9 datasets with the UFIDs for the 9 datasets are: 629e3d2c63914e45b227cc913c09cebe, 35524a031525484dab3b06f3728c708e, a957b431a74a43a0bb7cc52e1c84c8ad, 71f29913f174456e9fe2727b1b86b8b3, fda655aeb8644c9db5c543ed9d1006ad, 05fdeea9fcb64b3885e6ebfb85b4ce90, 1c565ac309074f178a377c2759333209, b73beac2f4c349fb981880399d4c88a6, d5bd8e4814904c58a79d7cdcd7c2a1bb.

We downloaded “x.csv” and “scaling.tar.gz” files in the same folder, then we uncompressed “scaling.tar.gz” and the “scaling” folder has “param.csv” and two folders “factuals” and “counterfactuals”. With a specific UFID, say “629e3d2c63914e45b227cc913c09cebe” for example, we used “629e3d2c63914e45b227cc913c09cebe.csv” from “factuals” folder and

“629e3d2c63914e45b227cc913c09cebe_cf.csv” from “counterfactuals” folder.

# Part 2: Code

## Abstract

We open sourced the all the code for data loading, models implementation, model training, and model evaluation at <https://github.com/liuq-lab/bayesgm> . Besides, we provide Pypi python standalone Package and built a website <https://causalbgm.readthedocs.io> for instructions and tutorials.

## Description

### Code format(s)

Script files

R  Python  Matlab

Other:

Package

R  Python  MATLAB toolbox

Other:

Reproducible report

R Markdown  Jupyter notebook

Other:

Shell script

Other (described here):

### **Supporting software requirements**

Version of primary software used

Python 3.9

Libraries and dependencies used by the code

TensorFlow 2.10.10; TensorFlow Probability 0.18.0, scikit-learn 1.6.1, pandas 2.3.3, numpy 1.26.4

### Supporting system/hardware requirements (optional)

### Parallelization used

No parallel code used

Multi-core parallelization on a single machine/node

Number of cores used:

Multi-machine/multi-node parallelization

Number of nodes and cores used:

### License

MIT License (default)

BSD

GPL v3.0

Creative Commons

Other (described here):

### Additional information (optional)

# Part 3: Reproducibility workflow

## Scope

The provided workflow reproduces:

Any numbers provided in text in the paper

The computational method(s) presented in the paper (i.e., code is provided that implements the method(s))

All tables and figures in the paper

Selected tables and figures in the paper, as explained and justified here:

Tables and figures contain results from competing methods. We only provide code and pipeline to reproduce the results of our CausalBGM approach, which are detailed below.

## Workflow details

### Format(s)

Single master code file

Wrapper (shell) script(s)

Self-contained R Markdown file, Jupyter notebook, or other literate programming approach

Text file (e.g., a readme-style file) that documents workflow

Makefile

Other (more detail in 'Instructions' below)

### Instructions

Overview of CausalBGM workflow

A typical end-to-end run consists of:

1. Data preparation: format the observed data into `(X, Y, V)` as required by the implementation (treatment/exposure `X`, outcome `Y`, and high-dimensional covariates `V`).

2. (Optional) initialization with EGM: an initialization stage to stabilize subsequent Bayesian iterative updates.

3. Iterative model fitting: train CausalBGM by repeatedly updating parameters and latent feature posteriors for a user-specified number of epochs.

4. Causal effect estimation with uncertainty: obtain point estimates and posterior intervals by posterior sampling (e.g., MCMC-based sampling in the provided implementation) at user-chosen significance level `α`.

Code structure

The implementation is organized as a Python package with:

`src/`: source code for models, datasets, configuration files, and runnable scripts.

`src/main.py`: an executable entry point that runs the full pipeline (loading data/configs → optional initialization → training → prediction/evaluation).

`bayesgm.models`: contains the CausalBGM model class and related model components.

`bayesgm.datasets`: built-in simulation datasets and dataset utilities used for experiments.

`bayesgm.utils`: utility functions to support CausalBGM training and testing.

`src/configs/`: YAML configuration files specifying model architecture, training hyperparameters.

Additionally, we provide

`doc/`: source code for the website <https://causalbgm.readthedocs.io/>, including a Makefile, Markdown files and the python notebook files for tutorials.

This design supports both (i) script-based reproduction via `main.py`under `src` folder and (ii) library usage via direct import of the `CausalBGM` class.

We provide two supported ways to reproduce the experimental results reported in the paper.

Option A — Run the provided script (`main.py`)

Users can reproduce results by running the end-to-end script located at `src/main.py`. The script reads a YAML configuration file (in `src/configs/`) and executes the full workflow:

* load/generate the dataset,

* Initialize the model (optional EGM stage),

* fit CausalBGM with the iterative Bayesian updating algorithm,

* compute causal estimands and posterior intervals.

A typical usage pattern is:

* select the configuration file corresponding to the experiment,

* run `python src/main.py -c path_to_config` (or the equivalent command described in the documentation),

* collect outputs (estimated effects, uncertainty intervals, and evaluation summaries) written by the script.

Option B — Install the package and use the API

Alternatively, users may install the released package and run experiments through the Python API. After installation, the workflow is:

* import `CausalBGM`,

* construct/load data `(X, Y, V)`,

* call `egm_init(...)` (optional but recommended),

* call `fit(...)`,

* call `predict(...)` to obtain point estimates and posterior intervals.

Importantly, we provide Jupyter Notebook ` tutorial_py.ipynb ` to help reproduce the results in both binary treatment setting and continous treatment setting. If users directly run `tutorial_py.ipynb`, the results for Imbens et al dataset in Figure 2 and Table 1 for CausalBGM are reproduced. The results for ACIC 2018 first dataset in Table 2 are reproduced. Users could easily reproduce the results for other datasets by simply changing the dataset loader. For binary treatment setting, users could replace “Sim_Hirano_Imbens_sampler” with “Sim_Sun_sampler”, “Sim_Colangelo_sampler”,” Semi_Twins_sampler” for reproducing results for other datasets. For continuous treatment setting, users could specify the Ufid parameter in “Semi_acic_sampler”.

Expected run-time

Approximate time needed to reproduce the analyses on a standard desktop machine:

<1 minute

1-10 minutes

10-60 minutes

1-8 hours

>8 hours

Not feasible to run on a desktop machine, as described here:

### Additional documentation (optional)

# Notes (optional)
