# Supplementary material for: An AI-powered Bayesian Generative Modeling Approach for Causal Inference in Observational Studies
Source: J Am Stat Assoc. Author manuscript; Available in PMC 2026 Jul 9. (PMC13344347; doi:10.1080/01621459.2026.2654227)
Supplement: Supp 1 [file NIHMS2185026-supplement-Supp_1.zip › online_supplements (9).zip!/online_supplements/CausalBGM_SI.pdf]

Appendix: An AI-powered Bayesian generative modeling approach for causal inference in observational studies

1 Appendix A: Proof of identifiability of $\mu(x)$

We aim to show that the average dose-response function $\mu(x) = \mathbb{E}[Y(x)]$ is identifiable under Assumption 1. Let Z_0 denote the latent confounding variable that affects both treatment X and oucome Y. We assume treatment X and oucome Y are related by two unknown functions:

$$\begin{cases} X = h_0(Z_x, U_1), \\ Y = f_0(X, Z_y, U_2), \end{cases} \tag{1}$$

where $U = (U_1, U_2)$ denote all unobserved variables, including disturbances. Z_x and Z_y are two latent representations of the original high-dimensional covariates V. Then, the latent confounding variable Z_0 is naturally defined as the intersection of the two feature sets Z_x and Z_y. We further assume additive error terms for (1) in our generative modeling.

$$\begin{aligned} \mu(x) &= \mathbb{E}[Y(x)] \\ &= \int \mathbb{E}[f_0(x, Z_y, U_2)|Z_0 = z_0)]p_{Z_0}(z_0)dz_0 \\ &= \int \mathbb{E}[f_0(X, Z_y, U_2)|X = x, Z_0 = z_0]p_{Z_0}(z_0)dz_0 \\ &= \int \mathbb{E}[Y|X = x, Z_0 = z_0)]p_{Z_0}(z_0)dz_0, \end{aligned} \tag{2}$$

where $p_{Z_0}(z_0)$ denotes the marginal distribution of latent confounding variable Z_0, the second equality is obtained by the law of total expectation and the third equality is obtained by Assumption 1 ($X \perp\!\!\!\perp Y(x)|Z_0$).

2 Appendix B: Parametric Setting of Generative Models

Our model learns the conditional distribution of observed variables given latent variables, where the conditional mean and variance functions are both learned by flexible neural networks to capture complex nonlinear relationships, offering greater flexibility and expressiveness. In default, we model the conditional distribution as normal distributions for continuous variables and logistic regression for discrete variables. Note that we do not directly assume the high-dimensional data (e.g., covariates) to be normally distributed. The default normal setting is imposed conditionally, not marginally. The distributional assumption (e.g., Gaussian) primarily affects the modeling of the conditional variance, not the mean. Therefore, even if the true data-generating process deviates from a multivariate Gaussian distribution, the learned mean function remains robust.

CausalBGM is designed to be extremely flexible, which can accommodate any parametric family. For example, in a Generalized Linear Model (GLM), we typically have

$$\begin{cases} \mathbb{E}(V|Z) = \mu = g^{-1}(Z\theta), \\ Var(V|Z) = V(g^{-1}(Z\theta)) \end{cases} \tag{3}$$

where $g(\cdot)$ is the link function and θ is the model parameter to be learned. In CausalBGM framework, we can further generalize GLM by replacing the linear term $Z\theta$ with a neural net that takes the latent variable Z as input. This allows us to model both mean and variance flexibly across different distributional families, including those in the exponential family.

3 Appendix C: Details of Datasets

Imbens et al

We follow a similar data-generating process as in Hirano and Imbens (2004) and Moodie and Stephens (2012) as follows: let V_1, V_2,...,V_p be i.i.d. unit exponential random variables, $Z_0 = V_1$, $Z_1 = V_2$, $Z_2 = V_3$, $X|V \sim exp(Z_0{+}Z_1)$, and $Y(x)|V \sim N(x{+}(Z_0{+}Z_2)exp(-x(Z_0{+}Z_2), 1)$. Then the dose-response function can be obtained by integration w.r.t the covariates V: $\mu(x) = x + \frac{2}{(1+x)^3}$.

Sun et al

We generate a synthetic dataset using a similar data generating process described in Sun et al. (2015). with some modifications to fit for continuous treatment. Specifically, we let $V_1, ..., V_p \overset{iid}{\sim} N(0, 1)$ and define $f_1(u) = -2sin(2u)$, $f_2(u) = u^2 - \frac{1}{3}$, $f_3(u) = u - \frac{1}{2}$, $f_4(u) = cos(u)$, $f_5(u) = u^2$ and $f_6(u) = u$. We then generate the treatment to be $X \sim N(\sum_{i=1}^{4} f_i(V_i), 1)$ and the outcome to be $Y \sim N(X + f_3(V_1) + f_4(V_2) + f_5(V_5) + f_6(V_6), 1)$. Then the dose-response function can be obtained by integration w.r.t the covariates V: $\mu(x) = x + 0.5 + e^{-0.5}$..

Lee et al

We followed a similar data generation process in Colangelo and Lee (2020) as follows: let $\epsilon_1 \sim N(0, 1)$, $\epsilon_2 \sim N(0, 1)$. The covariates are generated by $V = (V_1, ..., V_p)' \sim N(0, \Sigma)$ where $diag(\Sigma) = 1$ and $\Sigma_{i,j} = 0.5$ for $|i - j| = 1$. The treatment is generated by $X = \Phi(3V'\theta) + 0.75\epsilon_1 - 0.5$ where $\theta_j = 1/j^2$. The outcome is generated by $Y = 1.2X + 1.2V'\theta + X^3 + XV_1 + \epsilon_2$. The dose-response function is $\mu(x) = 1.2x + x^3$.

Twins

This dataset contains data of 71,345 twins, including their weights (used as treatment), mortality, and 50 other covariates (so $p = 50$) derived from all births in the USA between 1989-1991. Similar to Li et al. (2020), we first filtered the data by limiting the weight to be less than 2 kilograms. 4821 pairs of twins were kept for further analysis. We set the weights as the continuous treatment variable. We then simulate the risk of death (outcome) under a model in which higher weight leads to a lower death rate in general. Let Y be the Bernoulli variable where $Y = 1$ indicates death and R be the death risk that depends on the covariates. We simulate the outcome as $Y(x) \sim Bernoulli(R(x))$, $R(x) = -\frac{2}{1+e^{-3X}} + v\gamma + \epsilon$ where $\gamma \in \mathbb{R}^{p\times 1}$ and $\gamma_i \sim N(0, 0.025^2)$, $\epsilon \sim N(0, 0.25^2)$. Taking the response variable as R instead of Y, the ADRF is then: $\mu(x) = -\frac{2}{1+e^{-3x}} + \mathbb{E}[V_i \cdot \gamma]$.

ACIC 2018

For binary treatment settings, we downloaded the datasets from the 2018 Atlantic Causal Inference Conference (ACIC) competition. This dataset utilizes the Linked Births and Infant Deaths Database (LBIDD) based on real-world medical measurements collected from Karavani et al. (2018). The LBIDD data is semi-synthetic where 117 measured covariates are given, and the treatment and outcome are simulated based on different data-generating processes. We chose nine datasets by selecting the most complicated generation process (e.g., the highest degree of generation function) with sample sizes ranging from 1,000 to 50,000. The details for the datasets used in the study are provided in Table 1.

Table 1: Details of binary treatment datasets from ACIC 2018 used in this study. Each dataset has a unique ufid series number.

Sample size	Ufid	Treated percentage	True ATE
Datasets-1k	629e3d2c63914e45b227cc913c09cebe	36.76%	0.006208
	35524a031525484dab3b06f3728c708e	10.01%	0.513605
	a957b431a74a43a0bb7cc52e1c84c8ad	46.44%	6.373162
Datasets-10k	71f29913f174456e9fe2727b1b86b8b3	57.98%	9.243389
	fda655aeb8644c9db5c543ed9d1006ad	22.05%	-0.0566
	05fdeea9fcb64b3885e6ebfb85b4ce90	12.08%	0.025841
Datasets-50k	1c565ac309074f178a377c2759333209	14.48%	-0.468357
	b73beac2f4c349fb981880399d4c88a6	18.79%	-0.049168
	d5bd8e4814904c58a79d7cdcd7c2a1bb	54.50%	-0.296505

4 Appendix D: Baseline Methods

For the continuous treatment setting, three different baselines were used.

Ordinary Least Squares regression (OLS)

OLS first fit a linear regression model for $Y|(X, V)$. For each value of treatment x, the estimated ADRF is then $\frac{1}{n}\sum_i^n ls(x, v_i)$ where ls is the fitted linear model.

Regression Prediction Estimator (REG)

See Schafer and Galagate (2015); Galagate (2016); Imai and Van Dyk (2004). The *prima facie* estimator is an estimator that regresses the outcome on the treatment without considering covariates. REG generalizes the notion of *prima facie* estimator. It takes the covariates into account when doing regression. Unlike OLS, REG fits a quadratic ADRF: $Y(x) = \alpha_0 + \alpha_1 x + \alpha_2 x^2$.

Double Debiased Machine Learning Estimator (DML)

See Colangelo and Lee (2020). DML is a kernel-based machine learning approach that combines a doubly moment function and cross-fitting. Various machine learning methods can be used to estimate the conditional expectation function and conditional density. We used "Lasso", "random forest", and "neural network" provided by the DML toolkit as three variants, denoted as DML(lasso), DML(rf) and DML(nn).

For the binary treatment setting, six baselines were introduced.

CFR

See Shalit et al. (2017). This method estimated the individual treatment effect (ITE) by utilizing neural networks to learn the low-dimensional representation for covariates and two outcome functions, respectively. An integral probability metric was further introduced to control the balance of distributions in the treated and control group. We use the two variants of CFR for comparison, which are referred to as TAENET and CFRNET.

Dragonnet

See Shi et al. (2019). This method used a three-head architecture, which contains a two-head architecture for outcome estimation and a one-head architecture for propensity score estimation. It is noted that Dragonnet uses essentially the same architecture as CFR if the propensity-score head is removed.

CEVAE

See Louizos et al. (2017). This method is a variational autoencoder-based method for estimating the treatment effect where the above CFR architecture (Shalit et al., 2017) was

used in the inference network and the latent variables were set to be multivariate normal distribution.

GANITE

See Yoon et al. (2018). This method exploited a generative adversarial network (GAN) model for generating the counterfactual outcome through adversarial training.

CausalForest

.See Wager and Athey (2018). This method built random forests to estimate the heterogeneous treatment effect that is applicable in binary treatment settings. Causalforest is an ensemble method that consists of multiple causal trees.

CausalEGM

See Liu et al. (2024). CausalEGM is a deep learning framework designed to address challenges in causal inference within observational studies. It focuses on high-dimensional covariates by performing dependency-aware dimension reduction and generative modeling. CausalEGM employs a bidirectional transformation between high-dimensional covariates and a low-dimensional latent space, identifying latent features that influence both treatment and response. By conditioning on these features, the method mitigates confounding effects, enhancing causal effect estimation for both binary and continuous treatments. Theoretical analyses confirm its consistency and generalization capabilities.

SDRcausal

See Ghosh et al. (2021). This method uses linear sufficient dimension reduction for both treatment and outcome model. The propensity score is modeled as $1/(1+exp(-\eta(\alpha^T V)))$ and the potential outcome is modeled as $Y_k = m_k(\beta_T x) + \epsilon_k (k = 0, 1)$. α, β_0, and β_1 are the projection matrix/vector in the sufficient dimension reduction to be learned. Note that SDRcausal implements several different variants from the original paper and we always choose the best result to report.

5 Appendix E: Comparison with Sufficient Dimension Reduction

We made a comprehensive comparison of CausalBGM with SDRcausal under experimental settings either satisfying or violating the SDR assumption. CausalBGM shows great improvement over SDRcausal, especially in the nonlinear dataset.

We used the default hyperparameters of CausalBGM and used the default parameters provided by SDRcausal software. Note that SDRcausal implements several different variants from the original paper and we always chose the best result to report. We used two datasets (linear SDR and nonlinear SDR) for benchmark comparison.

1) The simulation dataset was obtained from the original SDRcausal paper, which satisfies the SDR assumption (linear). 6 covariates were considered. $V_1 \sim N(1,1), V_2 \sim N(0,1), V_3 \sim Bernoulli(0.5 + 0.005V_2), V_4 \sim 0.015V_1 + U(-0.5, 0.5), V_5 \sim Bernoulli(0.4 + 0.2V_4), V_6 = 0.004V_2 + 0.15V_3 + 0.05V_4 + N(0,1)$. $\alpha = (-0.27, 0.2, -0.15, 0.05, 0.15, -0.1)^T$,

$\beta_0 = (1, 1, 0, 0, 0, 0)^T$, $\beta_1 = (1, -1, 1, -2, -1.5, 0.5)^T$ The generation process is

$$\begin{cases} Y_0 = \beta_0^T V + N(0, 0.2), \\ Y_1 = 0.7(\beta_1^T V)^2 + \sin(\beta_1^T V) + N(0, 0.5), \\ P(X = 1|V) = 1/(1 + exp(-\alpha^T V)). \end{cases} \tag{4}$$

2) We replaced the linear SDR term with different nonlinear functions, such as polynomial and trigonometric terms, in the generation process of the first dataset, which is represented as

$$\begin{cases} Y_0 = m_0(V) + N(0, 0.2), \\ Y_1 = 0.7(m_1(V))^2 + \sin(m_1(V)) + N(0, 0.5), \\ P(X = 1|V) = 1/(1 + exp(-m_x(V))), \end{cases} \tag{5}$$

where $m_0(V) = \sin(V_1) + \cos(V_2) + \exp(V_3) + V_4^3 + \sin(V_5 V_6)$, $m_1(V) = \cos(V_1 V_2) + V_3^2 + \exp(V_4) + \sin(V_5) + \cos(V_6)$, $m_x(V) = V_1 V_2 + V_3 X_4^3 + V_5^2 V_6$. The linear SDR does not work in this case while the deep learning model CausalBGM can capture the nonlinearity in this dataset.

Then, we run CausalBGM and SDRcausal for independent 10 times for each dataset with different sample size. We used EGM initialization for 200,000 mini-batches. CausalBGM achieves significantly better performance than SDRcausal with default settings in both linear and nolinear settings with different sample sizes (Table 2). Even if we initiate the linear SDR parameters $(\alpha, \beta_0, \beta_1)$ to be the ground truth parameters in the generation process, CausalBGM could achieve slightly better performance than SDRcausal in terms of ϵ_{ATE}. Beside, we also found SDRcausal is not scalable as it fails to run on the ACIC datasets used in our paper when the sample size is larger than 10k.

Table 2: The comparison of CausalBGM and SDRcausal under both linear and nolinear SDR settings with three different sample sizes (2k, 5k, 10k). SDRcausal(DF) denotes using the default initial values in the model fitting. SDRcausal(GT) denoted directly giving the ground true parameter values as the initial values in the fitting of the model. Each method was run 10 times and the standard deviations are shown. The best performance is marked in bold.

Metric	Setting	SDRcausal(DF)	SDRcausal(GT)	CausalBGM
ϵ_{ATE}	Linear SDR	0.537 ± 0.070	0.034	$\mathbf{0.032 \pm 0.021}$
		0.415 ± 0.044	0.083	$\mathbf{0.072 \pm 0.028}$
		0.331 ± 0.044	0.038	$\mathbf{0.034 \pm 0.036}$
	Nolinear SDR	0.204 ± 0.131	-	$\mathbf{0.092 \pm 0.025}$
		0.176 ± 0.122	-	$\mathbf{0.010 \pm 0.013}$
		0.200 ± 0.185	-	$\mathbf{0.084 \pm 0.016}$
ϵ_{PEHE}	Linear SDR	14.49 ± 1.34	**0.684**	0.887 ± 0.076
		13.55 ± 1.39	**0.708**	0.782 ± 0.021
		13.31 ± 1.08	**0.711**	0.811 ± 0.032
	Nonlinear SDR	11.52 ± 0.58	-	$\mathbf{1.31 \pm 0.06}$
		11.13 ± 0.67	-	$\mathbf{1.21 \pm 0.09}$
		11.95 ± 1.08	-	$\mathbf{1.11 \pm 0.08}$

6 Appendix F: Scalability

For the scalability analysis, we are interested in 1) whether CausalBGM can handle datasets with large sample sizes; and 2) whether CausalBGM can handle datasets with a large number of covariates. We designed the following experiments to test the scalability of CausalBGM. For the continuous treatment experiment, we selected Hirano and Imbens dataset. We first change the number of covariates from 50 to 10000 while the sample size is 10000. Note that only OLS, DML(lasso) and CausalBGM can handle covariates more than 10,000 while other comparison methods failed (Figure 1 A). Next, we fix the number of covariates to 100 while changing the sample size from 10^3 to 10^6. Only OLS, REG, DML(lasso), and CausalBGM are able to handle large sample size 10^6 (Figure 1 B). Except for a small sample size situation (e.g., 1000) where CausalBGM achieves comparable performance compared to DML(nn) and DML(rf), CausalEGM consistently outperforms all comparison methods by either changing the number of covariates or sample size. Note that we benchmarked all methods using a Linux-based compute platform where the memory usage for each method is limited to 100 GB and running time is limited to 2 days in the scalability experiments.

7 Appendix G: Running Time

we conducted a series of computational time analyses using the Hirano and Imbens dataset with 200 covariates, varying the sample size across four orders of magnitude: 1,000, 10,000, 100,000, and 1,000,000. For each sample size, we recorded the total training time of CausalBGM under the same Linux environment with a single A100 GPU (80 Gb memory). The running time is limited to 2 days to ensure a reasonable time of a single experiment. The

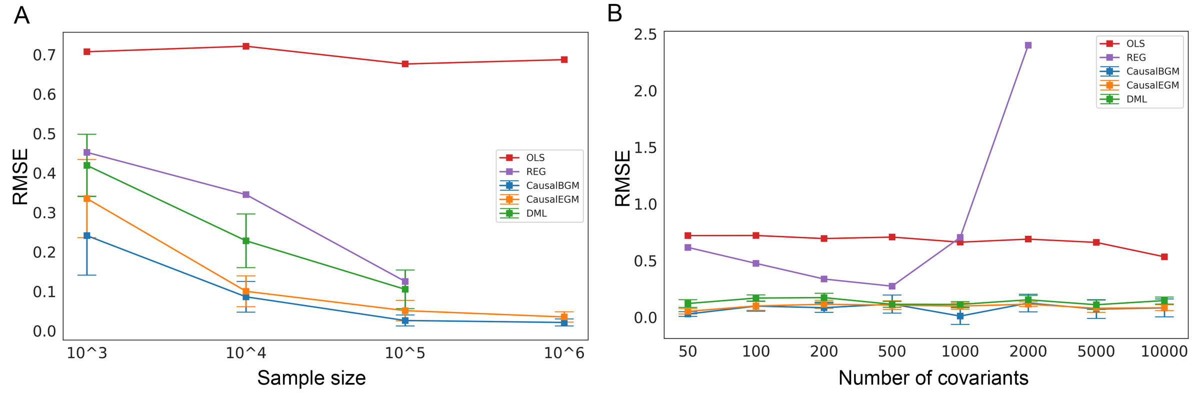

Figure 1: Scalability of CausalBGM model in terms of sample size and number of covariates. The error bar denotes the standard deviation of 10 independent runs. (A) Performance when changing the sample size from 10^3 to 10^6 in Hirano and Imbens dataset. (B) Performance when changing the number of covariates from 50 to 10000 in Hirano and Imbens dataset.

training takes around 28 minutes for 1,000 samples and 39 hours for 1M samples as shown in Figure 2. Theoretically, our CausalBGM can scale up to infinity samples single both steps in the iterative algorithm either requires the current sample or requires a mini-batch of samples in the iterative updating algorithm. Such design also guarantees the superior scalability for CausalBGM, making it a suitable causal inference tool in modern data science.

8 Appendix H: Identifiability

The identifiability requires that the joint distribution of the latent variables and observed variables can be uniquely identified from the marginal distribution of the observed variables under certain conditions. Identifiability of the latent variable model is notoriously difficult to directly achieve. In our case, the generative models for covariates, treatment,

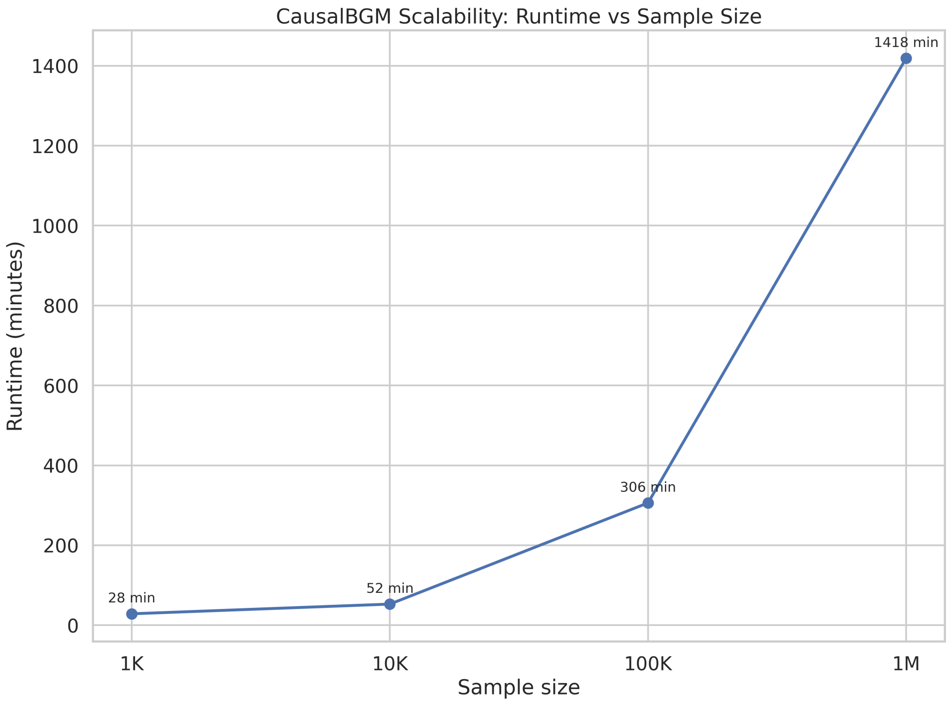

Figure 2: Running time of CausalBGM model on Imbens et al dataset with 200 covariates, given different sample sizes. The sample size varies from 10^3 to 10^6 in different magnitudes.

and outcome are parameterized by Bayesian neural networks, which learn both the conditional mean and variance functions. However, without additional constraints, the joint distribution of latent and observed variables may not be uniquely identifiable. For example, one can apply a rotation or nonlinear transformation to the latent variables and adjust the generative functions accordingly, resulting in the same marginal distribution over observed data.

To address the identifiability problem, we leverage the recent advances from nonlinear Independent Component Analysis (ICA) theory Hyvarinen and Morioka (2016, 2017); Hyvarinen et al. (2019), and iVAE framework Khemakhem et al. (2020), which show that identifiability can be achieved under mild conditions by introducing an auxiliary variable U and conditioning the latent prior on it.

To incorporate these ideas, we further developed an identifiable version of our model, iCausalBGM, which modifies the prior over latent variables to be conditionally Gaussian

$$Z|U \sim N(Z|\mu_{prior}(U), \sigma^2_{prior}(U)I). \tag{6}$$

where U is an auxiliary variable that we introduce by dividing the dataset into M equal-sized segments and assigning each sample a segment label $U \in \{1, ..., N\}$. U is uniformly distributed and $(\mu_{prior}, \sigma^2_{prior})$ is represented by a small neural network f_{prior} that maps the segment label U to the parameters of the Gaussian latent prior. Compared to the original CausalBGM, , iCausalBGM replace all the prior distribution of $P(Z)$ (e.g., standard Gaussian distrbution) with $P(Z|U)$ as defined in (6). Such conditioning breaks the symmetry of the latent space and enables identifiability up to trivial transformations, such as permutation and pointwise nonlinearities. The key intuition is that conditioning the latent prior on an auxiliary variable U, structured variation is introduced to the latent space to prevent arbitrary rotations or transformations of the latent variables from being indistinguishable.

This additional constraint ensures that the latent variables are recoverable up to simple transformations.

We implemented iCausalBGM and evaluated its performance using the Imbens et al. dataset, which contains continuous treatment and high-dimensional covariates. We randomly divided the dataset into equal-size $M = 10$ segments and used the segment label as the auxiliary variable U. The conditional prior network f_{prior} adopts a fully connected neural network with one hidden layer and 64 hidden nodes. We compared the performance of iCausalBGM to the original CausalBGM and other baselines, which is shown in Table 3.

Table 3: The performance of iCausalBGM, CausalBGM, and other baselines on Imbens et al. dataset. Root mean square deviation (RMSE) and mean absolute percentage error (MSPE) are used as evaluation metrics. Each method was run ten times.

Dataset	Method	RMSE	MAPE
Imbens et al	OLS	0.680 ± 0.0	0.367 ± 0.0
	REG	0.525 ± 0.0	0.214 ± 0.0
	DML (Lasso)	0.090 ± 0.0	0.037 ± 0.0
	DML (NN)	0.133 ± 0.022	0.052 ± 0.011
	CausalEGM	0.041 ± 0.014	0.019 ± 0.006
	CausalBGM	0.028 ± 0.007	0.019 ± 0.006
	iCausalBGM	0.034 ± 0.018	0.021 ± 0.013

It can be seen that iCausalBGM achieves slightly lower performance than the original CausalBGM but still very competitive among other baselines. Such results imply that incorporating the structured variation by auxiliary variable U enhances identifiability without compromising too much for the core strengths of CausalBGM. Such trade-off is acceptable in settings where interpretability and robustness of the latent space are critical.

9 Appendix I: Full MCMC implementation and comparison

In our CausalBGM framework, uncertainty arises from three distinct components: 1) Latent Space Uncertainty. This reflects the posterior distribution over latent variables Z, which is inferred for each individual. We use MCMC sampling (Metropolis-Hastings) to capture this uncertainty, ensuring that the posterior over Z is well-represented. 2) Generative Model Parameter Uncertainty. The parameters of the generative models G, H, F are treated as random variables and represented by Bayesian neural networks. This part explains the variation in the model space. 3) Conditional Variance in Final Sampling. Each generative model outputs both a mean and a variance function, allowing us to sample from the predictive distribution and capture residual uncertainty in the observed data. Together, these components allow CausalBGM to construct posterior intervals that reflect both epistemic and aleatoric uncertainty.

To investigate the impact of variational inference (VI) for interpreting uncertainty in model space. We develop a new model CausalBGM-MCMC, in which both the latent variables and model parameters are sampled via Markov Chain Monte Carlo (MCMC). Specifically, We use Hamiltonian Monte Carlo (HMC) to sample the weights of each generative model G, H, F. HMC leverages gradient information to efficiently to enable a more efficient sampling process. We set the burn-in steps to be $1,000$ and retrain $3,000$ samples per model for the model weights. In causal effect estimation, we randomly sample model weights from the HMC chains and latent variables from Metropolis-Hastings.

We compared the CausalBGM-MCMC with CausalBGM on the Imbens et al. dataset with sample size 5,000. For the causal effect estimation as shown in Table 4. The perfor-

mance of CausalBGM-MCMC is slightly lower than the original CausalBGM.

For posterior interval, we checked the interval length given a user-specific significance level α for average-dose response function $\mu(x)$ at 20 values uniformly distributed from $[0, 3]$. To our surprise, CausalBGM-MCMC achieves narrower interval than the original CausalBGM in 19 out of 20 values. We hypothesize that this may be due to the complexity and high dimensionality of the target distribution over neural network weights. The posterior landscape of model space is highly multimodal and difficult to explore thoroughly. Despite using HMC, the sampler may not fully capture the tails of the distribution, leading to underestimated uncertainty.

Table 4: The performance of CausalBGM (original) and CausalBGM-MCMC on Imbens et al. dataset with sample size 5,000. Root mean square deviation (RMSE) and mean absolute percentage error (MSPE) are used as evaluation metrics. Each method was run ten times.

Dataset	Method	RMSE	MAPE
Imbens et al	CausalBGM	0.037 ± 0.008	0.019 ± 0.004
	CausalBGM-MCMC	0.039 ± 0.007	0.023 ± 0.006

While CausalBGM-MCMC provides a more complete Bayesian setting, it comes with significant computational cost. In our experiments, CausalBGM-MCMC could only handle Imbens et al. dataset with 200 covariates, fewer than 10,000 samples on a NVIDIA RTX 2080 Ti GPU with 11 GB memory. This limitation arises because sampling model parameters via HMC requires evaluating the full likelihood, which scales poorly with sample size. In contrast, sampling latent variables in CausalBGM only requires access to the current sample and is independent of other samples, making it highly scalable. Our original CausalBGM framework can handle datasets with tens of millions of samples, making it far more suitable for modern large-scale applications in genomics, healthcare, and social

sciences.

10 Appendix J: Robustness to unmeasured covariates

CausalBGM relies on a latent-variable version of the unconfoundedness assumption (Assumption 1), which presumes that all confounding information can be captured by the observed covariates (or equivalently by the learned latent confounder). In practice, however, relevant covariates may be omitted or unmeasured. This appendix empirically evaluates how CausalBGM behaves under imperfectly measured covariates, with emphasis on which types of missing covariates lead to bias in causal effect estimation.

The impact of a missing covariate depends on its causal role. If an unmeasured covariate affects *only* the treatment X or *only* the outcome Y, it does not introduce confounding and therefore should not systematically bias causal effect estimation (though it may mildly reduce predictive accuracy). In contrast, if an unmeasured covariate affects both X and Y (i.e., a true confounder), then unmeasured confounding is introduced and the estimated causal effect can be biased.

We conducted a simulation study using the Imbens et al. dataset (the same setting as in the main text), and created three "unmeasured-covariate" scenarios by removing one known covariate from the observed covariate set and replacing it with random noise. This preserves the input dimension while eliminating the information carried by the removed covariate. We considered the following three scenarios:

1. **Unmeasured V_1 (affects only X):** remove a covariate that influences treatment X but not outcome Y.

2. **Unmeasured V_2 (affects only Y):** remove a covariate that influences outcome Y

Table 5: Robustness of CausalBGM to unmeasured covariates on the Imbens et al. dataset. Root mean square deviation (RMSE) and mean absolute percentage error (MSPE) are used as evaluation metrics.

Setting	RMSE	MAPE
Unmeasured V_1 (only affects X)	0.032	0.014
Unmeasured V_2 (only affects Y)	0.035	0.015
Unmeasured confounder V_0 (affects both X and Y)	0.082	0.023
Original (fully observed)	0.028	0.013

but not treatment X.

3. **Unmeasured confounder V_0 (affects both X and Y):** remove a covariate that jointly influences both X and Y.

For each scenario, we fit CausalBGM using the same modeling and evaluation pipeline as the original setting, and evaluated estimation accuracy of the causal estimand using the same metrics as in the main experiments: root mean squared error (RMSE) and mean absolute percentage error (MAPE).

Table 5 summarizes the performance of CausalBGM under the three unmeasured-covariate settings, together with the original (fully observed) setting. As expected, CausalBGM remains robust when the missing covariate affects only X or only Y, with only a slight loss of accuracy. In contrast, when a true confounder is unmeasured, we observe a clear degradation consistent with confounding-induced bias: RMSE increases substantially and MAPE increases as well. These results align with standard causal theory: CausalBGM cannot eliminate bias caused by truly unmeasured confounding without additional assumptions or auxiliary information. Practically, this experiment highlights that (i) including covariates that plausibly affect *both* treatment and outcome is critical.

References

Colangelo, K. and Y.-Y. Lee (2020). Double debiased machine learning nonparametric inference with continuous treatments. *arXiv preprint arXiv:2004.03036*.

Galagate, D. (2016). *Causal inference with a continuous treatment and outcome: Alternative estimators for parametric dose-response functions with applications.* Ph. D. thesis, University of Maryland, College Park.

Ghosh, T., Y. Ma, and X. de Luna (2021). Sufficient dimension reduction for feasible and robust estimation of average causal effect. *Statistica Sinica 31*(2), pp. 821–842.

Hirano, K. and G. W. Imbens (2004). The propensity score with continuous treatments. *Applied Bayesian modeling and causal inference from incomplete-data perspectives 226164*, 73–84.

Hyvarinen, A. and H. Morioka (2016). Unsupervised feature extraction by time-contrastive learning and nonlinear ica. *Advances in neural information processing systems 29*.

Hyvarinen, A. and H. Morioka (2017). Nonlinear ica of temporally dependent stationary sources. In *Artificial intelligence and statistics*, pp. 460–469. PMLR.

Hyvarinen, A., H. Sasaki, and R. Turner (2019). Nonlinear ica using auxiliary variables and generalized contrastive learning. In *The 22nd international conference on artificial intelligence and statistics*, pp. 859–868. PMLR.

Imai, K. and D. A. Van Dyk (2004). Causal inference with general treatment regimes: Generalizing the propensity score. *Journal of the American Statistical Association 99*(467), 854–866.

Karavani, E., Y. Shimoni, and C. Yanover (2018, January). Ibm causal inference benchmarking framework.

Khemakhem, I., D. Kingma, R. Monti, and A. Hyvarinen (2020). Variational autoencoders and nonlinear ica: A unifying framework. In *International conference on artificial intelligence and statistics*, pp. 2207–2217. PMLR.

Li, Y., K. Kuang, B. Li, P. Cui, J. Tao, H. Yang, and F. Wu (2020, 24 Aug). Continuous treatment effect estimation via generative adversarial de-confounding. In *Proceedings of the 2020 KDD Workshop on Causal Discovery*, Volume 127 of *Proceedings of Machine Learning Research*, pp. 4–22. PMLR.

Liu, Q., Z. Chen, and W. H. Wong (2024). An encoding generative modeling approach to dimension reduction and covariate adjustment in causal inference with observational studies. *Proceedings of the National Academy of Sciences 121*(23), e2322376121.

Louizos, C., U. Shalit, J. M. Mooij, D. Sontag, R. Zemel, and M. Welling (2017). Causal effect inference with deep latent-variable models. *Advances in neural information processing systems 30*.

Moodie, E. E. and D. A. Stephens (2012). Estimation of dose–response functions for longitudinal data using the generalised propensity score. *Statistical methods in medical research 21*(2), 149–166.

Schafer, J. and D. Galagate (2015). Causal inference with a continuous treatment and outcome: alternative estimators for parametric dose-response models. *Manuscript in preparation*.

Shalit, U., F. D. Johansson, and D. Sontag (2017). Estimating individual treatment effect:

generalization bounds and algorithms. In *International Conference on Machine Learning*, pp. 3076–3085. PMLR.

Shi, C., D. Blei, and V. Veitch (2019). Adapting neural networks for the estimation of treatment effects. *Advances in neural information processing systems 32*.

Sun, W., P. Wang, D. Yin, J. Yang, and Y. Chang (2015). Causal inference via sparse additive models with application to online advertising. In *Twenty-Ninth AAAI Conference on Artificial Intelligence*.

Wager, S. and S. Athey (2018). Estimation and inference of heterogeneous treatment effects using random forests. *Journal of the American Statistical Association 113*(523), 1228–1242.

Yoon, J., J. Jordon, and M. Van Der Schaar (2018). Ganite: Estimation of individualized treatment effects using generative adversarial nets. In *International Conference on Learning Representations*.
